# Weekly Image Guidance in Patients With Cervical Cancer Treated With Intensity‐Modulated Radiation Therapy: Results of a Large Cohort Study

**DOI:** 10.1002/cam4.70269

**Published:** 2024-10-01

**Authors:** Zheng Zeng, Weiping Wang, Junfang Yan, Dingchao Liu, Fuquan Zhang, Ke Hu

**Affiliations:** ^1^ Department of Radiation Oncology, Peking Union Medical College Hospital Chinese Academy of Medical Sciences & Peking Union Medical College Beijing China; ^2^ Department of Radiation Oncology, State Key Laboratory of Complex Severe and Rare Diseases, Peking Union Medical College Hospital Chinese Academy of Medical Science and Peking Union Medical College Beijing China

**Keywords:** cervical cancer, cone‐beam computed tomography, image‐guided radiotherapy, intensity‐modulated radiation therapy

## Abstract

**Background:**

Image guidance is recommended for patients undergoing intensity‐modulated radiation therapy (IMRT) for cervical cancer. In this study, we evaluated the feasibility of a weekly image guidance pattern and analyzed the long‐term outcomes in a large cohort of patients.

**Methods:**

The study enrolled patients with Stage IB‐IVA cervical cancer who received definitive radiotherapy or concurrent chemoradiotherapy. IMRT was delivered at a dose of 50.4 Gy in 28 fractions, with weekly cone‐beam computed tomography (CBCT). Physicians advised patients on rectum and bladder preparation to help them prepare on nonimaging guidance days. When significant tumor regression was observed, a second computed tomography simulation and replanning were performed.

**Results:**

The median follow‐up periods were 63.4 months. The incidence rates of loco‐regional and distant failure were 9.9% and 13.6%. The 5‐year overall survival (OS), disease‐free survival (DFS), loco‐regional relapse‐free survival (LRFS), and distant metastasis‐free survival (DMFS) rates were 80.1%, 72.9%, 78.3%, and 74.8%, respectively. For patients with different stages, the 5‐year OS, DFS, LRFS, and DMFS rates were statistically significant. For patients with and without positive regional lymph nodes, the 5‐year OS, DFS, LRFS, and DMFS rates were 64.5% and 86.0%, 56.8% and 78.8%, 62.7% and 84.3%, and 58.8% and 81.0%, respectively. Multivariate analysis showed that age, histology, tumor size, cancer stage, pretreatment squamous cell carcinoma antigen level, and para‐aortic metastatic lymph nodes were independent prognostic factors of OS. Fifty‐six (4.0%) patients experienced late Grade 3/4 chronic toxicities.

**Conclusions:**

IMRT with weekly CBCT is an acceptable image guidance strategy in countries with limited medical resources.

## 1 Introduction

Cervical cancer is the fourth most common malignancy among women worldwide, predominantly affecting developing countries, in which > 85% of cases are reported [1]. In China, cervical cancer has a high incidence of 10.86/100,000 and a mortality rate of 3.15/100,000 [2]. Radiotherapy plays a significant role in the treatment of cervical cancer, and approximately 80% of patients receive radiotherapy alone or in combination with chemotherapy [3].

The development of intensity‐modulated radiation therapy (IMRT) has been a major technical advancement in radiation therapy over the last two decades [4]. For patients with cervical cancer undergoing radiotherapy, IMRT can improve target conformity and reduce radiation exposure to healthy tissues such as the bowel, bladder, rectum, and pelvic bone marrow [5, 6]. IMRT reduces gastrointestinal and hematologic toxicities compared with three‐dimensional conformal radiation therapy [7, 8]. Inter‐ and intrafractional motion of organs at risk (OARs) may lead to underdosing of the target volume, which may affect patient survival [9]. The Comprehensive Cancer Network clinical guidelines indicate image‐guided radiotherapy (IGRT), including cone‐beam computed tomography (CBCT) or high‐energy megavoltage computerized tomography (MVCT) for patients with cervical cancer who receive IMRT [10]. IGRT is a technique that helps mitigate dose discrepancies resulting from variations in patient positioning, thereby minimizing the effects on the target volume and doses received by OARs [11]. Daily CBCT or MVCT is then recommended with IMRT. Most developing countries have a scarcity of essential medical resources, such as linear accelerators, despite the prevalence of cancer [12]. Weekly CBCT may be an alternative treatment option for these areas. This study analyzed the prognosis and toxicity of weekly CBCT in patients with cervical cancer who underwent definitive radiotherapy or concurrent chemoradiotherapy. Our results will help clinicians better guide treatment strategies for cervical cancer.

## 2 Methods

### 2.1 Patients

In this study, we reviewed patients with Stages IB–IVA cervical cancer treated with definitive IMRT at Peking Union Medical College Hospital from October 2005 to December 2015. All patients were confirmed for cervical cancer by pathology. Patients who received daily or weekly image guidance were included. Pretreatment evaluation included the essential diagnostic tests for gynecological physical examination, complete blood counts, liver and kidney function tests, urinalysis, squamous cell carcinoma antigen levels (SCC Ag), as well as chest and abdomen computed tomography (CT), pelvic magnetic resonance imaging (MRI), or positron emission tomography‐computed tomography (PET‐CT). Approval was obtained from the Institutional Review Board of Peking Union Medical College Hospital.

### 2.2 IMRT

Our previous studies comprehensively described the specific radiation approach [13, 14]. All patients were placed in the supine position for radiation therapy and were immobilized using a low‐temperature thermoplastic mask combined with a body board. The patients underwent CT using a 16‐slice Philips Brilliance Big Bore CT scanner with intravenous contrast agents. Before the simulation, the patients were instructed to ensure that their bladders contained approximately 250–350 mL of urine and to empty their rectum. Then, 10 mL of meglumine diatrizoate was mixed with 200–300 mL of water to be consumed 1–1.5 h before the simulation.

The gross tumor volume and clinical target volume (CTV) were determined based on imaging and clinical findings as described previously [15]. Metastatic lymph nodes (MLNs) were defined as lymph nodes with a short diameter (≥ 1 cm) on CT and MRI, or confirmed by PET‐CT. The regional MLNs were encompassed by gross tumor volume nodes (GTVnd). The CTV included the primary tumor, cervix, uterus, parametria, part of the vagina, and pelvic lymphatic drainage areas. Patients with positive common iliac or para‐aortic lymph nodes received expanded‐field radiotherapy (EFRT). If the primary tumor extended to the lower one‐third of the vagina, the CTV included the inguinal lymphatic area. Internal target volume was considered in this method. The planning target volume (PTV) was achieved by expanding the CTV by 0.7–1.0 cm in all directions, with an additional 0.5–1.0 cm of margin on the uterus. The planned gross tumor volume nodes (PGTVnd) consisted of a 0.5 cm outwardly expanded border from the GTVnd. The radiotherapy dose was delivered to the PTV using fixed‐field intensity‐modulated radiation therapy (FF‐IMRT) or volumetric volume‐modulated radiation therapy (VMAT). The prescribed dose consisted of a minimum of 95% planning clinical target volume, 50.4 Gy in 28 fractions, and the prescribed dose consisted of a minimum of 95% PGTVnd, and 56–60.2 Gy with a simultaneous integrated boost.

### 2.3 Image Guidance

The patients were required to empty their rectums and prepare their bladders before treatment, similar to the simulation. CBCT was performed every day or once weekly in the treatment position for all patients. The CBCT images were automatically matched to the planned CT by bony matching in three directions: left–right (LR), anterior–posterior (AP), and superior–inferior (SI). Experienced clinicians validated the matched images by making the necessary modifications. If an appropriate match was not achieved, the patient was repositioned and CBCT was repeated. Once a satisfactory match was obtained, the clinician assessed whether the PTV contained the primary tumor, uterus, cervix, and vagina. If these structures were not located within the PTV due to inadequate rectal or bladder preparation, radiation therapy was administered, and the patient was asked to repeat the rectal or bladder preparation. CBCT was performed again at the follow‐up, and the alignment process was repeated as described above. All measurement errors were corrected before treatment. Once a satisfactory match was achieved, radiation therapy was initiated. After treatment, physicians informed patients about the suitability of their bladder and rectum preparation. This feedback was intended to assist patients in preparing their bladder and rectum on nonimaging days. On these nonimaging days, patients were positioned based on skin markings.

To match tumor regression during treatment, a second CT simulation and IMRT planning session were performed for all patients. Typically, the second simulation and planning were performed after 20 fractions of external beam radiation therapy (EBRT) and 1–2 fractions of brachytherapy. If significant tumor regression was observed on CBCT images before the completion of 20 fractions, a second CT simulation was performed.

### 2.4 Brachytherapy

High‐dose‐rate brachytherapy typically commenced 3 weeks after IMRT using ^192^Ir as the radiation source. A prescribed dose of 30–36 Gy administered in 5–6 fractions was delivered at point A.

### 2.5 Chemotherapy

The chemotherapy regimen for concurrent therapy was selected based on individual circumstances. Patients who were frail, malnourished, or had contraindications to chemotherapy received radiotherapy alone. The most commonly used regimen was cisplatin‐based chemotherapy (30–40 mg/m^2^, weekly) administered during radiotherapy. Patients with renal dysfunction were administered paclitaxel (60–80 mg/m^2^) weekly.

### 2.6 Follow‐Up and Outcomes

As described in a previous study [13], the first follow‐up assessment was performed 1 month after the end of treatment. Subsequently, patients received regular checkups every 3 months for the first 2 years. Follow‐up assessments were performed every 6 months for 3–5 years and annually after 5 years. Routine follow‐up examinations included a gynecological physical examination, CT scans of the chest and abdomen, MRI or CT scans of the pelvis, and SCC Ag measurements. PET‐CT scans were used for patients with signs of recurrence or metastasis. Early radiation toxicity was assessed using the Common Terminology Criteria for Adverse Events version 3.0. Late radiation toxicity was evaluated using the Radiation Therapy Oncology Group Chronic Toxicity Response Evaluation Criteria.

### 2.7 Statistical Analysis

Statistical analysis was conducted using IBM SPSS Statistics for Windows, version 23.0 (IBM Corp, Armonk, NY, USA). Two‐sided *p*‐values ≤ 0.05 were considered statistically significant. The chi‐squared test was used to compare the baseline characteristics and toxicities among the patients in the different groups. The Kaplan–Meier method with log‐rank test was used to estimate overall survival (OS), disease‐free survival (DFS), locoregional relapse‐free survival (LRFS), and distant metastasis‐free survival (DMFS). Cox regression analysis was used for univariate and multivariate analysis of OS with medians and 95% confidence intervals. OS was defined as the time from cervical cancer diagnosis to death or last follow‐up. DFS was defined as the time from cervical cancer diagnosis to the occurrence of an event such as death; local, regional, or distant disease progression; or to the date of the last follow‐up. LRFS was defined as the time from cervical cancer diagnosis to the date of local/regional recurrence or the last follow‐up. DMFS was defined as the time from cervical cancer diagnosis to the date of distant metastasis or last follow‐up.

## 3 Results

### 3.1 Patient Characteristics

We collected data from 1395 patients with the 2018 International Federation of Gynecology and Obstetrics (FIGO) Stage IB–IVA cervical cancer treated with weekly IGRT. The patients' age ranged from 23 to 88 years, with a median age of 51 years. Patients' detailed clinical characteristics are summarized in Table 1. Among these patients, 1254 (91.9%) were diagnosed with squamous cell carcinoma. A total of 208 patients (14.9%) underwent PET‐CT; 401 (28.7%) patients had pelvic MLNs and 87 (6.2%) had para‐aortic MLNs. Stages IB, IIA, IIB, IIIA, IIIB, IIIC, and IVA cervical cancer was present in 130 (9.3%), 88 (6.3%), 559 (40.1%), 30 (2.2%), 132 (9.5%), 445 (31.9%), and 11(0.8%) patients, respectively. A total of 297 patients (21.3%) underwent EFRT. Cisplatin and paclitaxel chemotherapy regimens were administered to 1113 (79.8%) and 122 (8.7%) patients, respectively, with a median of five cisplatin cycles. In this study, 330 (23.7%) patients received > 8 weeks of treatment.

**TABLE 1:** Clinical characteristics of the patients.

Characteristics	No. (*N* = 1395)	Percentage
Age (years old)		
Median	51 (range, 26–88)	
< 65	1244	89.2
≥ 65	151	10.8
Histology		
Squamous cell carcinoma	1254	91.9
No squamous cell carcinoma	141	8.1
Tumor size (cm)		
< 4 cm	536	38.4
≥ 4 cm	859	61.6
Pretreatment SCC Ag (ng/mL)		
≤ 1.5	379	27.2
> 1.5	1016	72.8
FIGO stage (2018)		
IB	130	9.3
IIA	88	6.3
IIB	560	40.1
IIIA	30	2.2
IIIB	131	9.4
IIIC	445	31.9
IVA	11	0.8
Para‐aortic MLNs		
Yes	87	6.2
No	1308	93.8
Pelvic MLNs		
Yes	401	28.7
No	994	71.3
Cisplatin cycles		
Median	5	
No	160	11.5
1–4	471	33.7
≥ 5	764	54.8
EFRT		
Yes	297	21.3
No	1098	78.7
Treatment time (weeks)		
< 8	1065	76.3
≥ 8	330	23.7

Abbreviations: EFRT, extended‐field radiotherapy; FIGO, International Federation of Gynecology and Obstetrics; MLNs, metastatic lymph nodes; SCC Ag, squamous cell carcinoma antigen.

### 3.2 Patterns of Failure

A total of 377 (24.2%) patients experienced treatment failure, including locoregional failure (138 patients, 9.9%), distant failure (190 patients, 13.6%), and locoregional and distant failure (49 patients, 3.5%). Of the 286 (20.5%) patients who died, 31 (10.8%) died of noncervical cancer.

Fifty‐five of the patients with pelvic failure did not achieve complete remission. The most common sites of pelvic failure were the pelvic lymph nodes (42 patients), cervix (34 patients), vagina (32 patients), and parametria (15 patients). The rates of pelvic failure at 1, 2, 3, 4, 5, 6, 7, and 8 years were 10.0%, 16.4%, 19.1%, 20.9%, 21.7%, 22.1%, 23.1%, and 23.6%, respectively. Among extrapelvic failures, the common sites included the lungs (110 patients), supraclavicular lymph nodes (38 patients), bones (34 patients), para‐aortic lymph nodes (24 patients), liver (21 patients), and mediastinal lymph nodes (5 patients). The rates of extrapelvic failures at 1, 2, 3, 4, 5, 6, 7, and 8 years were 9.8%, 18.6%, 22.0%, 23.9%, 25.2%, 26.5%, 27.9%, and 29.1%, respectively.

### 3.3 Patient Survival

**FIGURE 1:**
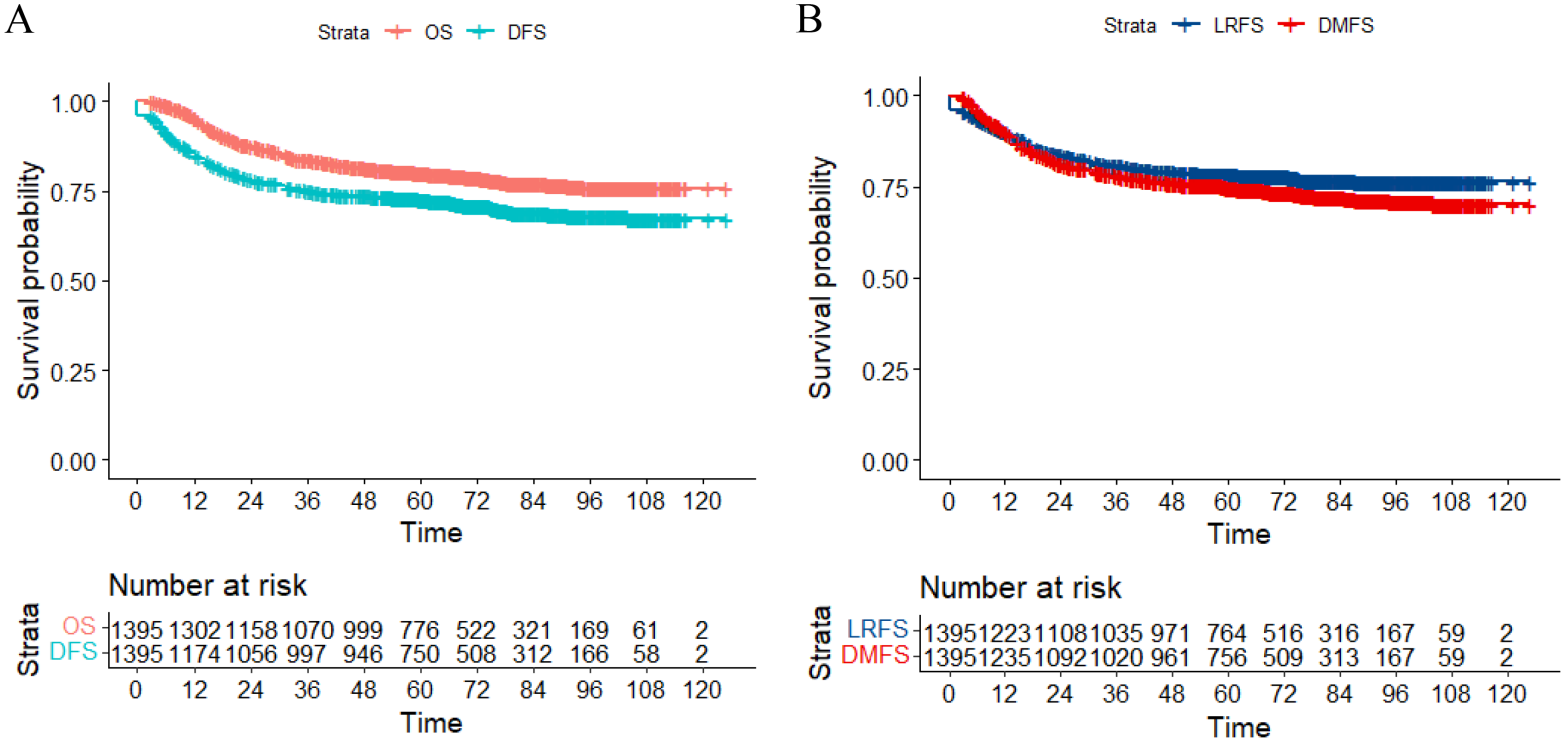
Survival rates of patients with cervical cancer treated with intensity‐modulated radiation therapy with weekly image guidance. (A) OS, DFS, (B) LRFS, and DMFS. DFS, disease‐free survival; DMFS, distant metastasis‐free survival; LRFS, locoregional relapse‐free survival; OS, overall survival.

**FIGURE 2:**
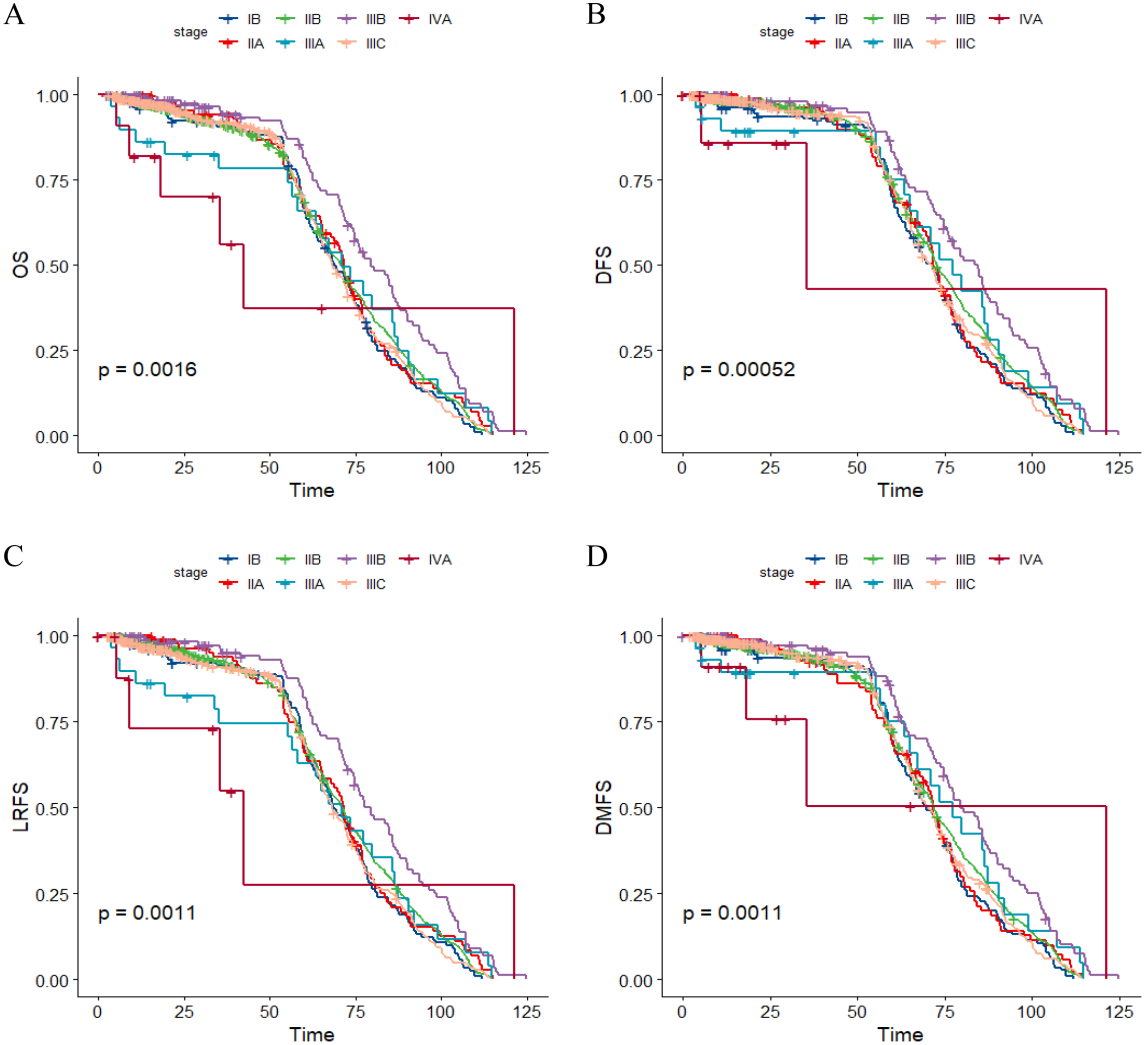
Survival rates of patients with different stages of cervical cancer. (A) OS, (B) DFS, (C) LRFS, and (D) DMFS. DFS, disease‐free survival; DMFS, distant metastasis‐free survival; LRFS, locoregional relapse‐free survival; OS, overall survival.

The median follow‐up period for all patients was 63.4 months (range 2.4–124.9 months). As shown in Figure 1, the 5‐year OS, DFS, LRFS, and DMFS rates were 80.1%, 72.9%, 78.3%, and 74.8%, respectively. The 5‐year survival rates for patients with Stages IB, IIA, IIB, IIIA, IIIB, IIIC, and IVA disease were 95.8%, 91.8%, 86.4%, 80.8%, 70.3%, 69.0%, and 48.6% (OS) (*p* = 0.0016); 91.2%, 85.0%, 78.5%, 71.0%, 66.0%, 60.6%, and 21.2% (DFS) (*p* = 0.00052); 93.5%, 89.7%, 84.2%, 84.9%, 69.8%, 67.1%, and 33.2% (LRFS) (*p* = 0.0011); and 92.0%, 88.4%, 81.3%, 71.0%, 66.5%, 62.5%, and 36.0% (DMFS) (*p* = 0.0011), respectively (Figure 2). The number of patients with and without regional MLNs were 410 and 985, respectively. In patients with and without regional MLNs, the 5‐year OS, DFS, LRFS, and DMFS rates were 64.5% and 86.0% (*p* < 0.001), 56.8% and 78.8% (*p* < 0.001), 62.7% and 84.3% (*p* < 0.001), and 81.0% and 58.8% (*p* < 0.001), respectively.

### 3.4 Analysis of Prognostic Factors

To determine the prognostic factors for OS in patients with cervical cancer treated with weekly IGRT, 1395 patients were analyzed based on a variety of clinicopathological characteristics. Univariate analysis was used to identify significant prognostic factors affecting OS. The significant prognostic factors identified in the univariate analysis were further evaluated using multivariate analysis (*p* < 0.05) (Table 2). The multivariate analysis using the Cox regression model showed that age, histology, tumor size, FIGO staging, pretreatment SCC Ag level, and para‐aortic MLNs were independent prognostic factors affecting OS.

**TABLE 2:** Univariate and multivariate analysis of OS in patients with cervical cancer.

Characteristics	Univariate analysis		Multivariate analysis	
	HR (95% CI)	*p*	HR (95% CI)	*p*
Age (years old)				
< 65	1		1	
≥ 65	1.72 (1.25–2.36)	< 0.001	2.22 (1.61–3.06)	< 0.001
Histology				
Squamous cell carcinoma	1		1	
No squamous cell carcinoma	1.50 (1.12–2.03)	0.007	1.51 (1.11–2.05)	0.009
Tumor size (cm)				
< 4	1		1	
≥ 4	2.26 (1.72–2.98)	< 0.001	1.74 (1.31–2.32)	< 0.001
Pretreatment SCC Ag (ng/mL)				
≤ 1.5	1		1	
> 1.5	1.78 (1.32–2.40)	< 0.001	1.49 (1.09–2.03)	0.011
FIGO stage				
IB	1		1	
IIA and IIB	2.29 (1.11–4.72)	0.025	2.07 (1.00–4.27)	0.050
IIIA–IVA	5.83 (2.86–11.84)	< 0.001	3.41 (1.61–7.23)	0.001
Pelvic MLNs				
No	1		1	
Yes	2.76 (2.19–3.48)	< 0.001	1.27 (0.90–1.79)	0.166
Para‐aortic MLNs				
No	1		1	
Yes	6.29 (4.70–8.42)	< 0.001	3.42 (2.47–4.75)	< 0.001
Cisplatin cycles				
≤ 4	1			
≥ 5	0.85 (0.68–1.08)	0.184		
Extended field irradiation				
No	1			
Yes	0.82 (0.61–1.10)	0.188		
Treatment time (weeks)				
< 8	1			
≥ 8	0.93 (0.70–1.23)	0.601		

Abbreviations: FIGO, International Federation of Gynecology and Obstetrics; MLNs, metastatic lymph nodes; OS, overall survival; SCC Ag, squamous cell carcinoma antigen.

### 3.5 Toxicities

The types and frequencies of acute toxicity events at all levels during treatment are shown in Table 3. The most common acute toxic reactions were hematological toxicities (particularly neutropenia and leukopenia). Grade 3/4 neutropenia occurred in 24.7% (345/1395) of patients, whereas Grade 3/4 leukopenia occurred in 41.0% (568/1395) of patients. Grade 3/4 anemia occurred in 10.5% (146/1395) of patients. Grade 3 gastrointestinal and genitourinary system toxicities occurred in 10.0% (139/1395) and 9.5% (133/1395) of patients, respectively. Only two patients developed Grade 4 gastrointestinal complications.

**TABLE 3:** Acute toxicity of IMRT with weekly image guidance in patients with cervical cancer.

Adverse events	Grade 1 no. (%)	Grade 2 no. (%)	Grade 3 no. (%)	Grade 4 no. (%)
Anemia	256 (18.4)	393 (28.2)	109 (7.8)	37 (2.7)
Neutropenia	181 (13.0)	324 (23.2)	296 (21.2)	49 (3.5)
Leukopenia	54 (3.9)	315 (22.6)	521 (37.3)	47 (3.7)
Thrombocytopenia	206 (14.8)	186 (13.3)	87 (6.2)	4 (0.3)
Vomit	111 (8.0)	260 (18.6)	95 (6.8)	0 (0)
Abdominal pain	427 (30.6)	90 (6.5)	20 (1.4)	0 (0)
Diarrhea	309 (22.2)	174 (12.5)	123 (8.8)	2 (0.1)
Rectitis	250 (17.9)	100 (7.2)	9 (0.6)	0 (0)
Genitourinary effects	214 (15.3)	78 (5.6)	133 (9.5)	0 (0)

Abbreviation: IMRT, intensity‐modulated radiation therapy.

Late Grade 3/4 complications occurred in 4.0% (56/1395) of patients, including three patients with late Grade 3/4 complications in both systems. Gastrointestinal side effects were observed in 2.6% (36/1395) of patients and 1.5% (21/1395) had genitourinary side effects. One patient developed hematologic complications, and another developed lower extremity edema.

### 3.6 Comparison

Compared with patients receiving weekly imaging guidance, patients treated with daily imaging guidance were in the more advanced stages of the disease (Table S1). The baseline distribution of histology, tumor size, FIGO stage, para‐aortic MLNs, pelvic MLNs, cisplatin cycles, EFRT, and treatment time differed among the patients included in the study. Survival analysis was performed, and propensity score matching (PSM) was conducted to compare weekly and daily IGRT. After 1:1 PSM, 37 patients were included in the weekly IGRT group and 37 patients in the daily IGRT group. In the overall study population, there was no significant difference in survival outcomes between the daily and weekly IGRT groups (5‐year OS 66.3% vs. 67.6%, *p* = 0.663; 5‐year DFS 56.3% vs. 64.5%, *p* = 0.712; 5‐year LRFS 75.2% vs. 67.6%, *p* = 0.414; and 5‐year DMFS 56.3% vs. 64.5%, *p* = 0.789). Furthermore, 13.5% (5/37) and 21.6% (8/37) of patients in the daily and weekly IGRT groups, respectively, developed acute Grade 3/4 gastrointestinal toxicity (*p* = 0.359). In addition, genitourinary system toxicity was observed in 5.4% (2/37) and 13.5% (5/37) of patients in the daily and weekly IGRT groups, respectively (*p* = 0.233). Intergroup differences in late toxicities, such as gastrointestinal and genitourinary effects, were not significant (*p* > 0.05).

## 4 Discussion

The results of the present study revealed that weekly imaging guidance is rational for patients with cervical cancer treated with IMRT. The 5‐year OS, DFS, LRFS, and DMFS rates were 80.1%, 72.9%, 78.3%, and 74.8%, respectively. Distant failures were observed more frequently (13.6%) than local failures (9.9%). Multivariate analysis demonstrated that age, histology, tumor size, FIGO stage, pretreatment SCC Ag, and para‐aortic MLNs were independent prognostic factors for OS. The incidence of acute and late complications associated with IMRT and weekly IGRT was low. There were no statistically significant differences in survival outcomes and complications between the daily and weekly IGRT groups.

Owing to the tumor and surrounding organ movements, deformations, and volume changes to ensure high precision, the application of margins to the CTV to generate the PTV is crucial [16]. In a previous study on three‐dimensional conformal radiation therapy plans, Lee et al. [17] proposed PTV margins for the entire CTV consisting of 15 mm in the SI direction, 18 mm in the lateral direction, and 23 mm in the AP direction, respectively. Moreover, previous studies also recommended applying margins of approximately 15–24, 11–15, and 7–16 mm in the AP, SI, and LR directions of IMRT, respectively [18]. IMRT is increasingly utilized to minimize gastrointestinal, genitourinary, and hematological toxicities. However, excessively large PTV margins must be avoided, as they can compromise the benefits of IMRT [8, 19].

IGRT is an essential modality for managing and minimizing treatment uncertainties, thereby reducing the chances of missing the target area or overdosing the OARs [20]. IGRT improves the precision of patient positioning, leading to better preservation of normal tissues using tighter margins [11]. Many IGRT systems are available in clinics, the most widely used of which is CBCT [21]. Patni et al. analyzed 105 patients with gynecological malignancies who underwent daily CBCT for FF‐IMRT/VMAT. A total of 2078 CBCT images were obtained. The margins of the planning target volume (M_PTV_) were 5.8, 10.3, and 5.6 mm in the AP, SI, and LR directions, respectively. Yao et al. [22] collected 573 daily CBCT scans of 13 patients with gynecologic cancers and reported M_PTV_ values of the initial interfraction errors of 5.6, 8.3, and 7.6 mm along the AP, SI, and LR directions, respectively. The results of the present study demonstrated that daily CBCT is a reliable approach for precise patient positioning in the treatment of gynecological cancers [23]. However, daily imaging guidance has the disadvantage of additional radiation dose. Using weekly CBCT scans, Wang et al. [24] revealed standard deviations of systematic errors of 0.21 mm, 0.55, and 0.08 mm in the LR, SI, and AP directions, respectively. The standard deviations of the random errors were 3.2, 3.5, and 2.8 mm, respectively. M_PTV_ values were 5.4, 7.2, and 5.9 mm in the LR, SI, and AP directions, respectively. After reasonable bladder and rectum preparation, the M_PTV_ was relatively small based on daily or weekly image guidance. A margin of 0.7–1.5 cm is recommended when an internal target volume is used, according to the Radiation Therapy Oncology Group consensus guidelines [25]. In our study, we used a rational margin to ensure the coverage of mostly the target volume in weekly imaging guidance.

Other imaging guidance methods have been reported. Collen et al. [26] collected 150 daily pretreatment MVCT scans of patients with cervical cancer and reported M_PTV_ values of the cervix of 17, 12, 9, 8, 15, and 9 mm in anterior, posterior, left, right, superior, and inferior directions, respectively. Besides, Taylor et al. [27] reported that 33 gynecological cancer patients underwent MRI scans on 2 consecutive days. They suggested an asymmetrical margin with an M_PTV_ of 15 mm in the AP direction, 15 mm in the SI direction, and 7 mm in the LR direction. Owing to the long scan durations and limited medical resources, daily MRI IGRT may be difficult for some patients. Chan et al. [28] assumed that internal tumor and organ motion by using weekly MRI scans; the results showed that the margins of cervix were 10–15 mm in all directions. Meanwhile, Wang et al. [24] suggested M_PTV_ values of 5.4, 7.2, and 5.9 mm in the LR, SI, and AP directions, respectively, by analyzing weekly CBCT scans. Compared with MVCT or MRI, weekly CBCT can help evaluate the motion of tumor and organ because it is fast and widely available. In addition, our previous study showed that weekly CBCT may be a rational imaging guidance strategy for patients with cervical cancer [29].

The reduction in margin implied a decreased dose to the OARs and the incidence of adverse exhibited a downward trend [30]. Yao et al. [22] reported that image guidance could reduce M_PTV_. Reducing the margin size allows the maximum dose reduction to the small intestine, bladder, and femoral head, as well as an average dose reduction to the rectum, small intestine, bladder, and pelvic marrow. IGRT in combination with IMRT may further decrease hematologic and digestive toxicities in patients with cervical cancer because the dose to the marrow and bowels can be safely reduced without compromising the target coverage [30, 31]. IGRT may not only reduce adverse effects but also improve prognosis. Image guidance during treatment was not used or described in some previous studies. Hasselle et al. [32] conducted a study on 81 patients who received definitive IMRT followed by brachytherapy. The 3‐year OS rate was 61.4%, and the DFS rate was 51.4%. The estimated incidence of late Grade 3 or higher toxicity was 7%. In addition, Chen et al. [33] conducted a retrospective study including 109 cervical cancer patients treated with IMRT. The study found that the 3‐year OS rate was 78.2%, whereas the 3‐year DFS rate was 67.6%. Late Grade 3 or 4 gastrointestinal and genitourinary side effects were observed in 4.5% and 7.3% of the patients, respectively. In our study, we included a large number of patients with longer a follow‐up time using weekly IGRT. The 5‐year OS and DFS rates were 80.1% and 72.9%, respectively. Late Grade 3 or 4 gastrointestinal and genitourinary complications occurred in only 2.6% and 1.5% of patients. Moreover, we did not find any statistically significant differences in survival and adverse reactions between the weekly and daily IGRT groups. However, the prognosis and toxicity of patients with cervical cancer who receive radiotherapy are affected by various factors such as cisplatin cycles and radiotherapy range [13, 34]. Prospective controlled trials are needed to confirm the benefits of IGRT.

Tumor regression can alter the positions of the organs and structures to a large extent during radiotherapy for cervical cancer [21]. Lee et al. [17] analyzed 17 patients with cervical cancer who received radical radiotherapy and reported a 50% reduction in tumor volume after receiving 30.8 Gy EBRT. Chen et al. [35] showed that the gross tumor volume decreased from 79.62 mL before treatment to 20.86 mL at the end of EBRT, whereas the CTV decreased from 672.59 to 608.26 mL. The cervical position changed significantly after 15 fractions of EBRT. Beadle et al. [36] assessed the extent of tumor regression and cervical mobility during concurrent chemoradiotherapy for cervical cancer. The average cervical volumes before and after receiving 45 Gy EBRT were 97.0 mL and 31.9 mL, respectively. The mean volume reduction was 62.3%. The regression observed in this study led to alterations in the cervical position. Moreover, changes in bladder filling significantly affect the shape and position of the cervix and uterus. Bondar et al. [37] showed a mean uterine motility of 4.2 mm for changes in bladder volume < 50 mL and 11.2 mm for changes > 50 mL. Considering tumor regression and organ motion, a second CT simulation appears to be crucial for targets exhibiting significant variability. At our institution, a second CT simulation and adaptive radiotherapy plans are usually performed after 36 Gy of EBRT and 1–2 fractions of brachytherapy.

Radiotherapy plays an indispensable role in the treatment of cervical cancer, and great progress has been made, driven by technological advances in image guidance [12]. IGRT may be even more relevant because of the shortage of radiotherapy resources, including linear accelerators, in most developing countries [38]. In our institution, a single accelerator efficiently meets the treatment needs of 100 patients per day. We successfully deliver definitive IMRT to > 300 patients with cervical cancer annually [14]. Weekly imaging guidance can save considerable medical resources. Survival was high and adverse reactions were low in patients who were treated with definitive IMRT with weekly CBCT.

However, some limitations of this single‐center retrospective study may have led to a selection bias. Secondly, two‐dimensional brachytherapy was administered in this study, which could have influenced the prognosis. In addition, FF‐IMRT and VMAT were not compared in patients also receiving weekly IGRT. Future trials to validate the advantages of weekly IGRT are warranted.

## 5 Conclusions

The results of this study demonstrated that weekly CBCT is a viable approach. Among patients with cervical cancer who underwent IMRT, the 5‐year OS, DFS, LRFS, and DMFS rates were 80.1%, 72.9%, 78.3%, and 74.8%, respectively. Notably, more instances of distant failures (13.6%) than local failures (9.9%) were observed. Age, histology, tumor size, FIGO stage, pretreatment SCC Ag level, and para‐aortic MLNs were independent prognostic factors for OS. The incidence rates of Grade 3 acute and late gastrointestinal complications associated with IMRT and weekly IGRT were 10.0% and 9.5%, and 2.6% and 1.5% for urogenital system complications, respectively. There was no statistically significant difference in survival outcomes and adverse effects between the patients receiving daily IGRT and the patients receiving weekly IGRT. The findings of this large cohort study with prolonged follow‐up provide valuable insights for future investigations into the appropriate IGRT techniques for cervical cancer.

## Author Contributions

**Zheng Zeng:** conceptualization (equal), data curation (equal), formal analysis (equal), writing – original draft (equal). **Weiping Wang:** data curation (equal), investigation (equal), writing – original draft (equal). **Junfang Yan:** supervision (equal). **Dingchao Liu:** data curation (equal). **Fuquan Zhang:** funding acquisition (lead), supervision (equal), writing – review and editing (equal). **Ke Hu:** conceptualization (equal), resources (equal), supervision (equal), writing – review and editing (equal).

## Ethics Statement

The studies involving human participants were reviewed and approved by the Institutional Review Board of Peking Union Medical College Hospital. Informed consent was obtained from all subjects involved in the study.

## Conflicts of Interest

The authors declare no conflicts of interest.

## Supporting information


Table S1.

## Data Availability

The datasets used and/or analyzed for the present study are available from the corresponding author upon reasonable request.
