# Supplementary material for: Weekly Image Guidance in Patients With Cervical Cancer Treated With Intensity‐Modulated Radiation Therapy: Results of a Large Cohort Study
Source: Cancer Med. 2024 Oct 1;13(18):e70269. doi: 10.1002/cam4.70269 (PMC11443159; doi:10.1002/cam4.70269)
Supplement: Supplementary file 1 — Table S1. [file CAM4-13-e70269-s001.docx]

**TABLE S1** Characteristics of patients with cervical cancer treated with weekly IGRT or daily IGRT

| Characteristics | Weekly IGRT (N=1395) | Daily IGRT (N=38) | P |
| --- | --- | --- | --- |
| Age （years old） |  |  | 0.953 |
| <65 | 1244 (89.2%) | 34 (89.4%) |  |
| ≥65 | 151 (10.8%) | 4 (10.6%) |  |
| Histology |  |  | 0.007 |
| Squamous cell carcinoma | 1254 (91.9%) | 29 (76.3%) |  |
| No squamous cell carcinoma | 141 (8.1%) | 9 (23.7%) |  |
| Tumor size (cm) |  |  | 0.012 |
| <4 cm | 536 (38.4%) | 7 (18.4%) |  |
| ≥4 cm | 859 (61.6%) | 31 (81.6%) |  |
| Pretreatment SCC Ag(ng/mL) |  |  | 0.054 |
| ≤1.5 | 379 (27.2%) | 5 (13.2%) |  |
| >1.5 | 1016 (72.8%) | 33 (86.8%) |  |
| FIGO stage (2018) |  |  | 0.001 |
| IB-IIB | 778 (55.8%) | 11 (28.9%) |  |
| IIIA-IVA | 617 (44.2%) | 27 (71.1%) |  |
| Para-aortic MLNs |  |  | <0.001 |
| Yes | 87 (6.2%) | 9 (23.7%) |  |
| No | 1308 (93.8%) | 29 (76.3%) |  |
| Pelvic MLNs |  |  | <0.001 |
| Yes | 401 (28.7%) | 25 (65.8%) |  |
| No | 994 (71.3%) | 13 (34.2%) |  |
| Cisplatin cycles |  |  | 0.021 |
| ＜5 | 631 (45.2%) | 10 (26.3%) |  |
| ≥5 | 764 (54.8) | 28 (73.7%) |  |
| EFRT |  |  | <0.001 |
| Yes | 297 (21.3%) | 32 (84.2%) |  |
| No | 1098 (78.7%) | 6 (15.8%) |  |
| Treatment time（weeks） |  |  | 0.003 |
| ＜8 | 1065 (76.3%) | 21 (55.3%) |  |
| ≥8 | 330 (23.7%) | 17 (44.7%) |  |

IGRT, image-guided radiotherapy; SCC Ag, squamous cell carcinoma antigen; FIGO, International Federation of Gynecology and Obstetrics; MLNs, metastatic lymph nodes; EFRT, extended-field radiotherapy.
